# Identification of four novel Streptomyces isolated from machair grassland soil using a culture-based bioprospecting strategy: Streptomyces caledonius sp. nov., Streptomyces machairae sp. nov., Streptomyces pratisoli sp. nov. and Streptomyces achmelvichensis sp. nov.

**DOI:** 10.1099/ijsem.0.006736

**Published:** 2025-04-09

**Authors:** Josephine R. Prole, Nick Allenby, David A.C. Manning, Michael Goodfellow

**Affiliations:** 1 School of Natural and Environmental Sciences, Newcastle University, Newcastle upon Tyne, UK; 2 John Dawson Drug Discovery Centre, Sunderland University, Sunderland, UK

**Keywords:** machair grassland soil, novel *Streptomyces* species, specialized metabolites, taxonomic description

## Abstract

A culture-dependent bioprospecting strategy, based on the use of several selective isolation media, revealed the presence of relatively high numbers of streptomycete-like colonies from machair grassland soil, in which carbonate minerals dominate. Representatives were shown to be bioactive in primary and secondary antimicrobial screens conducted through standard plug assays. The comparison of the whole-genome sequences showed that four of the isolates were novel species in the genus *Streptomyces*, for which the names *Streptomyces caledonius* sp. nov. (=DSM 118365; =NCIMB 15554), *Streptomyces machairae* sp. nov. (=DSM 118363; =NCIMB 15553), *Streptomyces pratisoli* sp. nov. (=DSM 118364; =NCIMB 15555) and *Streptomyces achmelvichensis* sp. nov. (=NCIMB 15556; =DSM 118366) are proposed. Genomes of the novel strains were found to be rich in biosynthetic gene clusters predicted to encode for diverse, specialized metabolites, notably antibiotics. They also contained stress-related genes that provided an insight into how streptomycetes cope with the prevailing conditions in machair grassland soils. It can be concluded that selective isolation and dereplication of streptomycetes from the unique machair habitat provides a practical way of isolating novel *Streptomyces* strains for ecological and biotechnological studies.

## Introduction

Natural products remain the predominant source of pharmaceutically interesting biomolecules needed to control multidrug-resistant microbial pathogens [1] and fungal phytopathogens [2] and to promote the growth of plants [3]. Filamentous actinomycetes classified in the phylum *Actinomycetota* [4] remain an important source of new antibiotics [5]. Novel streptomycetes are especially gifted in this respect as they have large genomes (>8.0 Mbp), which include biosynthetic gene clusters (BGCs), many of which encode for new and uncharacterized antibiotics [6,7]. Indeed, previously unknown streptomycetes are still the most promising source of chemically diverse, specialized (secondary) metabolites [8,9].

Extreme and neglected ecosystems are prolific sources of novel streptomycete species with the ability to synthesize new antibiotics [10,11], exemplified by *Streptomyces leeuwenhoekii* strains, which produce new antibacterial and antitumour compounds [12]. The genomes of gifted filamentous streptomycetes may also contain genus and species-specific BGCs [13,14], discoveries that emphasize the importance of sound classification in the search for isolates for bioprospecting surveys, especially ones focussed on taxonomic approaches to drug discovery [15,16]. Similarly, genome-based classifications of novel filamentous streptomycetes facilitate the choice of gifted representatives for bioprospecting and ecological studies [16,17].

Neglected habitats, still to be the subject of taxonomic surveys, include the unique machair grassland soils, which are mainly found in coastal areas of Northwest Scotland and the Outer Hebrides [18]. In the present study, isolates of streptomycete-like colonies, from selective isolation plates inoculated with mineral particles of machair grassland soil, were assigned to groups based on colonial pigment colours [16] and whole-genome sequence analyses carried out on representatives of the colour groups to determine taxonomic status. The antimicrobial activity and physiological properties of selected strains were determined using standard procedures. Finally, whole-genome sequences of novel strains were mined to determine the numbers and types of BGCs and stress-related genes. The overall objectives of the study were to determine the taxonomic diversity of streptomycetes from a typical machair grassland soil and their functional activities and to generate a strain library that can be used to address critical issues facing agricultural, industrial and medical biotechnology.

## Methods

### Physicochemical properties

Physicochemical properties of an environmental sample collected from the machair rhizosphere soil at Achmelvich Bay, Sutherland, Scotland (National Grid Reference NC 0570424848) [19] by Professor Michael Goodfellow on 25 May 2023 (Fig. S1, available in the online Supplementary Material) were determined. Their mineralogical composition was established using X-ray powder diffraction [20] and other physicochemical features established by standard procedures, as described by Álvaro-Fuentes *et al.* [21]. Thermogravimetry-differential scanning calorimetry quadrupole with Mass Spectrometry (TG-DSC-QMS) was performed to quantify the calcium carbonate content, using a Netzsch STA449C Jupiter TG-DSC-QMS system [22].

### Isolation of filamentous streptomycetes

Dry soil samples (1 g), pre-treated at 60 °C for an hour to reduce the number of non-sporulating, fast-growing bacteria, were sprinkled over 90 mm Millipore filters (0.45 µm pore size) and placed over various selective media: actinomycete isolation [23], arginine-vitamin [24], Gause’s [25], humic acid-vitamin [26], raffinose-histidine [27] and starch-casein vitamin agar [28] – supplemented with cycloheximide, nalidixic acid and nystatin [29] (each at 25 µg ml^−1^). Selective media were chosen from standard protocols for the isolation of actinomycetes so that their characterization would be comparable to type strains in reference collections. Five plates of each medium were incubated at 28 °C for 3 days when the filters were removed and discarded. The plates were then incubated for an additional 7 days, and the number of c.f.u. per gramme of dry weight soil growing on the selective media was recorded.

### Selection, dereplication and preservation of representative isolates

Representatives of filamentous colony types growing on the selective isolation plates were subcultured onto oatmeal agar [International *Streptomyces* Project (ISP) medium 3 [30]] and peptone-yeast extract-iron agar plates (ISP medium 6 [29]) and incubated at 28 °C for 14 and 7 days, respectively, for dereplication through colour grouping. Colour codes were taken from the ISCC-NBS charts [31].

Isolates representing the colour groups were maintained on actinomycete isolation agar at pH 8.1 and stored at 4 °C. Spores and mycelial fragments scraped from the actinomycete isolation agar plates, incubated for 7 days at 28 °C, were suspended in 20% (v/v) glycerol and stored at −80 °C for long-term preservation.

### Physiology

Seven isolates, representing the multi- and single-membered colour groups, were examined for their ability to grow at a range of pH values (5.5, 6.5, 7.5, 8.5 and 9.5), sodium chloride concentrations (10, 30, 50, 70 and 90 ppt) and temperatures (4, 15, 28, 37 and 45 °C) using starch casein-vitamin agar as the basal medium. Phosphate buffers were used to control the pH values; the pH and salinity plates were examined after 7-day incubation at 28 °C and the temperature plates after 7-day incubation. Gram staining was also performed following the protocol described by Smith and Hussey [32].

According to the updated guidelines of the International Code of Nomenclature of Prokaryotes or IJSEM for taxonomic description, chemotaxonomic analyses such as polar lipid and fatty acid analysis are no longer obligatory for the characterization of novel bacterial species. They are only mandated for novel genera [33].

### Antimicrobial screening

#### Primary screens

The same seven isolates were screened for antimicrobial activity against a panel of five WT strains *Escherichia coli*, *Bacillus subtilis*, *Pseudomonas fluorescens*, *Micrococcus luteus* and *Saccharomyces cerevisiae* using the standard agar plug assay described by Fiedler [34] onto Luria-Bertani agar plates. Plugs of the 15 representative isolates were cultivated on actinomycete isolation agar at pH 8.1 for 7 days at 28 °C and placed on the prepared assay plates, 6 per plate, incubated overnight at 37 °C after which any inhibition zones were recorded in millimetres.

#### Secondary screens

The isolates were grown on actinomycete isolation agar for 5 days at 28 °C prior to screening against five *B. subtilis* mutant reporter strains *yvqI*, *yvgS*, *ypuA*, *yjaX* and *dinB* using the standard agar plug procedure, to establish the mode of action of unknown bioactive compounds produced by representative isolates, as described by Kusuma *et al.* [35]. The *B. subtilis* mutants were grown overnight in 5 ml LB broth, and the resultant cultures were supplemented with 150 ml LB agar. The media were also supplemented with 40 mg ml^−1^ X-gal [36] and 50 mg ml^−1^ erythromycin or 34 mg ml^−1^ chloramphenicol, as shown in Table S1. All the preparations were incubated at 37 °C overnight, and the plates were checked for the presence of blue halos around inhibition zones. Blue halos appear when 5-bromo-4-chloro-3-hydroxyindole is formed as a consequence of X-gal cleavage due to the inhibition of bioactive products by the *B. subtilis* reporter genes [37].

### Genome sequencing

Genomic DNA was extracted using a Monarch® HMW DNA Extraction Kit by New England BioLabs®, according to the manufacturer’s instructions. To ensure the concentration and purity of extracted gDNA was adequate for sequencing, it was evaluated using 1% (w/v) agarose gel electrophoresis and NanoDrop™ spectrophotometer, as shown in Table S2. Seven isolates, representing each colour group, were sequenced using a MinION flow cell (R10.4.1) and Native Barcoding Kit 24 V14 from Oxford Nanopore Technologies. The sequencing was performed over 72 h to generate reads, and base calling was conducted using Guppy (version 6.4.2) [38]. Genomes were assembled into contigs using Flye (version 2.9.2) software [39], and 16S rRNA gene sequences were retrieved from whole genomes using Barrnap, which predicts the location and sequence of rRNA genes [40].

### Genomic comparison

Key genomic features of strains were determined using the RAST-SEED web server (https://rast.nmpdr.org/) [41,43] following the standard annotation procedures [44]. Average Nucleotide Identity (ANIb and ANIm) analyses and correlation indexes of TETRA-nucleotide signature were carried out using the JSpeciesWS server (https://jspecies.ribohost.com/jspeciesws) [45]. Additionally, dDNA:DNA hybridization (dDDH) *d*4 values were calculated using the Genome-to-Genome Distance Calculator 2.1 server (http://ggdc.dsmz.de; formula 2), according to the method described by Meier-Kolthoff *et al.* through the Type Strain Genome Server (TYGS) [46,47]. Scores were calculated to compare strains with their closest relatives. Strains were considered to belong to novel species if they shared dDDH, ANIb, ANIm and TETRA values with their closest phylogenomic neighbour below 70, 95, 95 and 0.99 respectively, following the established practice [48,50].

### Phylogeny

The intergenomic distances of the proposed novel strains and their closest phylogenomic neighbours, derived from TYGS [46,47], were used to infer a balanced minimum evolution tree with branch support via FASTME 2.1.6.1 including SPR postprocessing [51]. Branch support was inferred from 100 pseudo-bootstrap replicates each. The tree was rooted at the midpoint [52] and visualized with PhyD3 [53].

The obtained 16S rRNA gene sequences were compared with entries in public databases using blastn (https://blast.ncbi.nlm.nih.gov/Blast.cgi) with the default settings. To ensure well-characterized and taxonomically validated reference strains, the 16S rRNA sequences were only blasted against type strains. Phylogenies were derived by the maximum-likelihood method using the mega 11 software package [54]. Evolutionary distances were calculated using the correction from the Tamura–Nei model [55]. Bootstrap analysis of the maximum-likelihood data using 1,000 resamplings was carried out to determine the confidence in the branching points [56].

### Genomic mining

The presence of BGCs in the genomes of novel strains was detected using antiSMASH, version 7 (https://antismash.secondarymetabolites.org/). AntiSMASH detects and predicts BGCs by aligning regions at the gene cluster level to their closest neighbours from a database of all currently known secondary metabolite BGCs [57,58].

## Results and discussion

### Physicochemical properties

It is evident from the physicochemical properties analyses that the pH and conductivity of the soil sample are high, the phosphate and organic carbon contents are relatively low and the total inorganic content is high (Table 1). Similar results were reported by Rennert and Herrmann [18] who noted that low levels of phosphate were associated with high pH. The mineral fraction is dominated by carbonate minerals (Fig. S2) derived from broken marine mollusc shells: calcite and aragonite (both CaCO_3_), with minor amounts of magnesian calcite [Ca,Mg(CO_3_)], indicated by the high inorganic carbon value shown in Table 1. Quartz (SiO_2_) is also present.

**Table 1.** Physicochemical properties of the machair environmental sample

pH	Conductivity (μS cm^−1^)	Phosphate (mg g^−1^)	CaCO_3_ (wt%)	Total organic carbon (wt%)	Total inorganic carbon (wt%)
8.98	114.4	0.02	78.39	0.82	9.53

### Isolation of filamentous actinomycetes

**Fig. 1.**
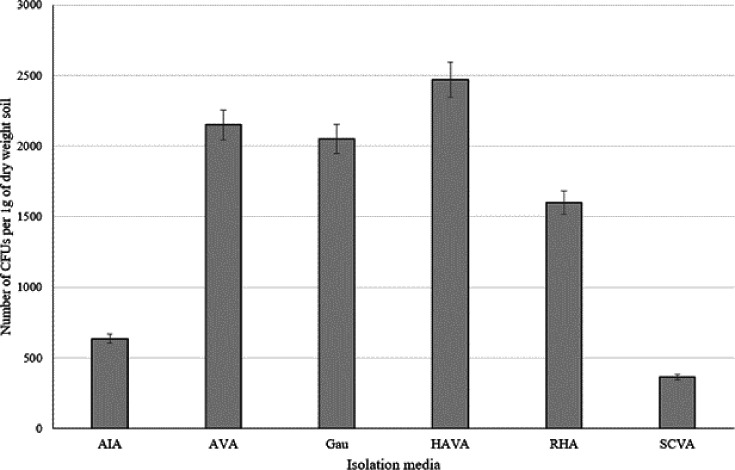
Number of c.f.u. of filamentous actinomycetes growing on actinomycete isolation agar (AIA), arginine-vitamin agar (AVA), Gause’s agar (Gau), humic acid-vitamin agar (HAVA), raffinose-histidine agar (RHA) and starch-casein vitamin agar (SCVA). Error bars represent 5% sd.

It is evident from Fig. 1 that across the selective media chosen, the lowest number of c.f.u. was recorded on the actinomycete isolation and starch-casein vitamin agar plates and the highest c.f.u. on the corresponding arginine-vitamin, Gause’s and humic acid-vitamin plates.

### Selection, dereplication and preservation of representative isolates

All representative colonies taken from the isolation plates were found to be filamentous actinomycetes as they produced leathery colonies, covered in many cases by aerial hyphae; such colonies are characteristic of streptomycetes. Twenty-one representative isolates taken from the arginine-vitamin (eight isolates), Gause’s (three isolates), humic acid (five isolates), raffinose-histidine (one isolate) and starch casein-vitamin (four isolates) plates were assigned to five multi-membered (two to eight isolates) and two single-membered colour groups. This was based on aerial spore mass colour, substrate mycelium colour and diffusible pigment colours produced on the ISP3 plates [59,60] and on the production of melanin pigments on ISP6, as shown in Table S3.

### Physiological characteristics

All the isolates grew well at 15 and 28 °C, all but two and three at 4 and 37 °C, respectively, as shown in Table 2 and Fig. S3, and none of the isolates grew at 45 °C. Each of the isolates grew well in the presence of 10 and 30 ppt NaCl, less well at 50 ppt NaCl and relatively poorly, if at all, at the two higher salinity values (Table 2 and Fig. S4). These growth patterns are consistent with most species of the *Streptomyces* genus. Remarkably, nearly all the isolates grew well at each pH though the best growth across the pH range was at the higher values (Table 2 and Fig. S5). The growth observed at pH 9.5 is notable as the optimum pH for *Streptomyces* is reported at pH 6.5–8.0 [61].

**Table 2.** Ability of isolates to grow over a range of pH, temperature and salinity values following incubation on starch casein-vitamin agar for 7 days

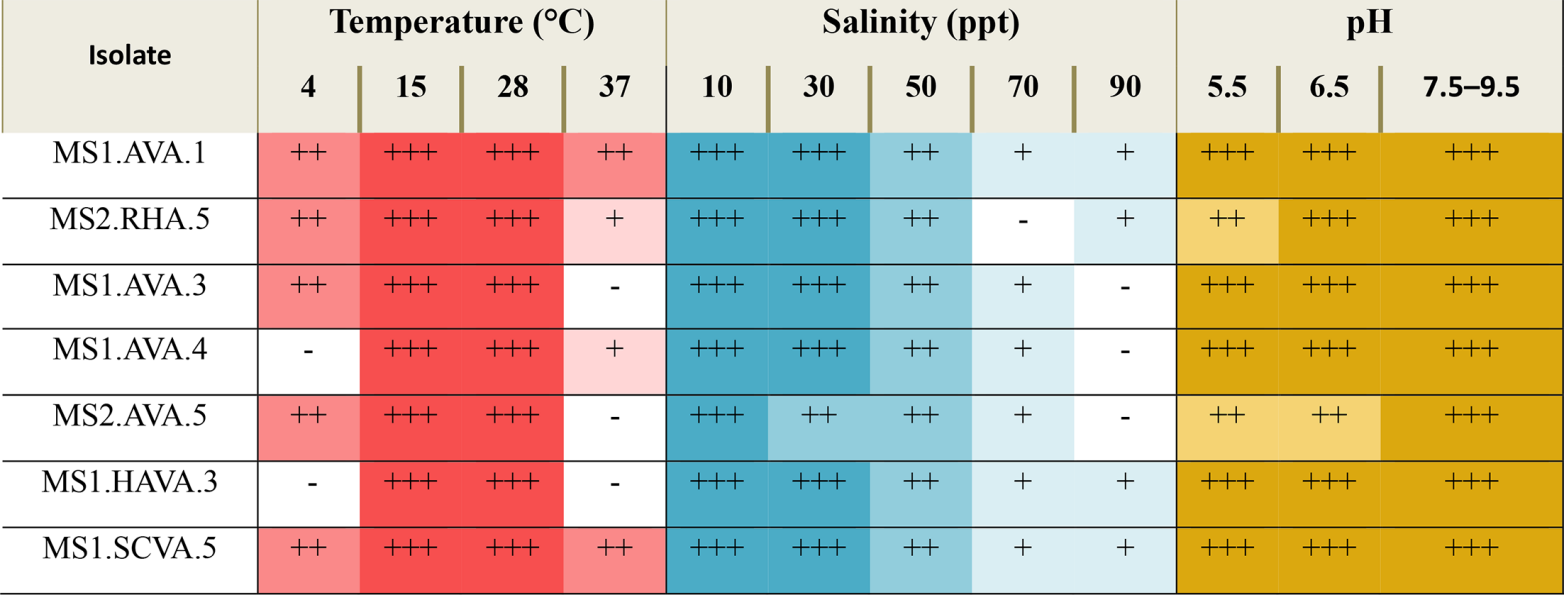 ­

+++, Good growth; ++, moderate growth; +, weak growth; -, no growth.

### Antimicrobial screening

#### Primary screens

Most of the isolates inhibited the growth of the *Saccharomyces cerevisiae* (71.4%) and *M. luteus* (57.1%) strains, whereas fewer inhibited the growth of the *B. subtilis* strain (42.9%) (Table S4). In contrast, none of the isolates inhibited the growth of the *E. coli* strain, and only one isolate, MS1.SCVA.5, inhibited that of the *P. fluorescens* strain.

#### Secondary screens

It is noteworthy that the majority of isolates inhibited the growth of *B. subtilis* mutants without forming blue halos (Table S5), indicating the production of bioactive compounds targeting cellular pathways not associated with halo formation (Table S1). This suggests that these isolates may produce compounds, which may interact with uncharacterized targets within the cells. A particularly notable isolate, MS1.AVA.3, produced blue halos against multiple *B. subtilis* mutants (*yvqI*, *yvgS*, *ypuA* and *dinB*), suggesting it generates secondary metabolites that interact with specific gene products or pathways.

### Genomic comparison

Key genomic properties of the isolates are shown in Table 3. All the isolates have large genomes ranging from 8.1 (isolate MS1.SCVA.5) to 9.8 Mbp (isolate MS1.AVA.3), that is within the range expected of streptomycetes, namely, 5.18 to 11.9 Mbp [62,63]. The corresponding dDNA G+C values fell within a narrow range, namely, 70.04% (isolate MS2.AVA.5) to 73.3% (isolate MS1.SCVA.5), expected by members within the genus *Streptomyces* [64]. Similarly, the number of protein coding sequences ranged from 7,031 to 11,297, the number of RNA operons from 75 to 92, N50 values from 705,683 to 9,329,654 and L50 values from 1 to 4.

**Table 3.** General genomic features of isolates, representing the colour groups

Isolate	Assembly size (bp)	No. of contigs	G+C content (%)	N50	L50	Protein coding sequences	No. of RNA operons
MS1.AVA.1	9419317	2	70.54	9329654	1	11,297	84
MS2.RHA.5	8413997	2	71.29	8170817	1	7,671	88
MS1.AVA.3	9833046	30	70.6	2302348	2	9,536	87
MS1.AVA.4	8612186	4	70.79	8456076	1	8,033	84
MS2.AVA.5	9630876	41	70.04	8403191	1	9,234	89
MS1.HAVA.3	8849353	6	71.79	7431408	1	10,757	92
MS1.SCVA.5	8079921	29	73.3	705683	4	7,031	75

N50, the length of the shortest contig for which longer and equal length contigs cover at least half of the assembly length; L50, count of the smallest number of contigs where the length sum accounts for the genome size.

**Fig. 2.**
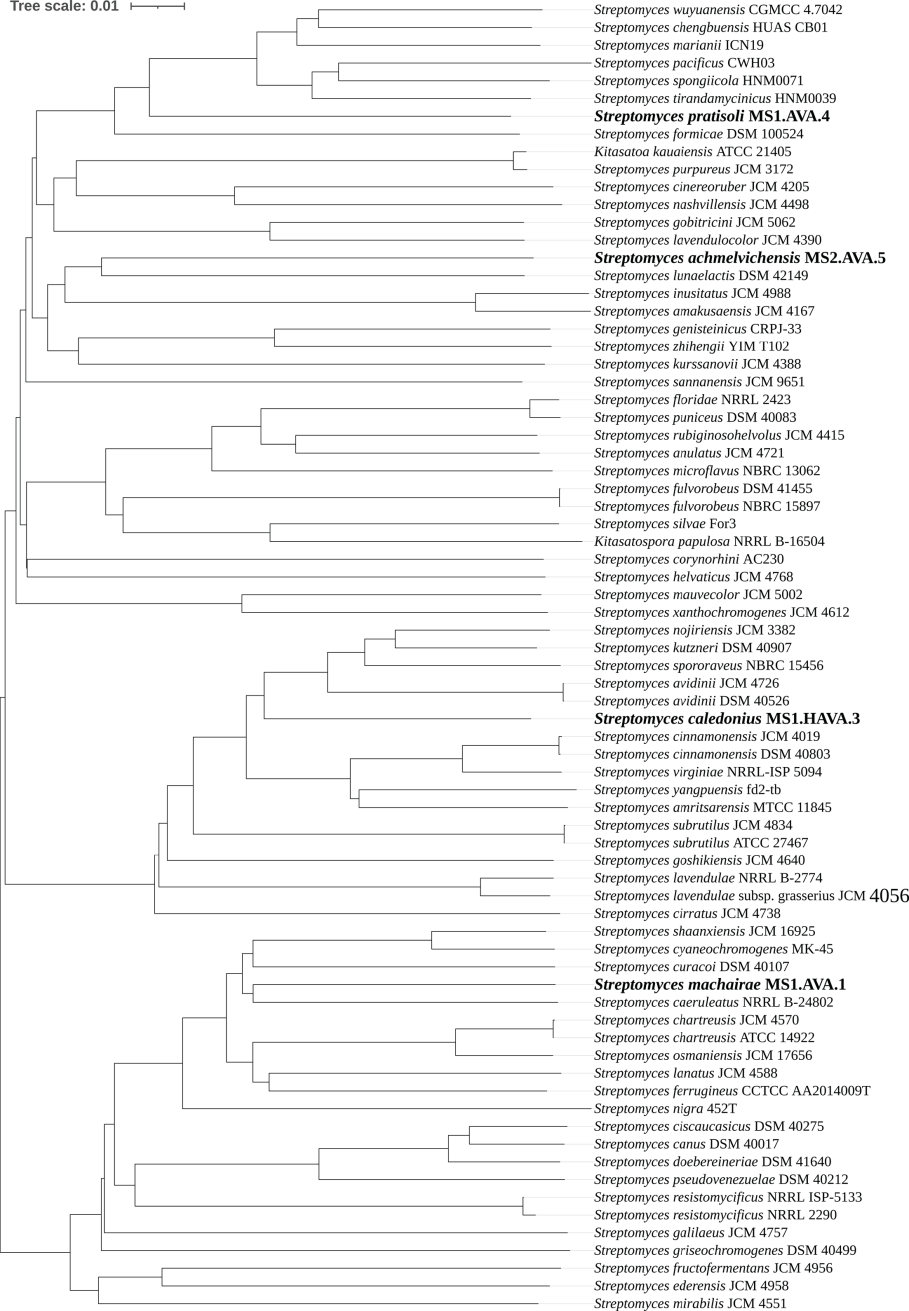
Phylogenomic tree based on whole-genome sequences of *Streptomyces achmelvichensis*, *Streptomyces caledonius*, *Streptomyces machairae* and *Streptomyces pratisoli*. The branch lengths are scaled in terms of GBDP distance formula *d*_5_. The numbers above branches are GBDP pseudo-bootstrap support values >60% from 100 replications, with an average branch support of 91.8%. The tree was rooted at the midpoint.

The dDDH, ANIb, ANIm and TETRA similarities found between the isolates and between them and their closest type strain neighbours are shown in Table 4. It is evident that on this basis, isolate MS1.SCVA.5 can be considered an authentic strain of *Streptomyces cacaoi* [65,66] given the dDDH, ANIb, ANIm and TETRA similarity of 91.3, 99.0, 99.0 and 0.99975, respectively, between the isolate and type strain of this species, values well above the cut-off point for grouping closely related strains to the same species. Similarly, isolate MS2.RHA.5 can be assigned to *Streptomyces microflavus* [67,68] given the dDDH, ANIb, ANIm and TETRA similarity of 76.5, 96.6, 97.4 and 0.99975, respectively, and isolate MS1.AVA.3 was assigned to *Streptomyces decoyicus* [69] with dDDH, ANIb, ANIm and TETRA values of 65.8, 95.7, 95.7 and 0.99947, respectively. Strains identified as *Streptomyces cacaoi*, *Streptomyces microflavus* and *Streptomyces decoyicus* have been shown to be active against breast cancer cell lines [66] and a range of pathogenic micro-organisms [68]. In contrast, the four remaining strains can be considered to belong to novel *Streptomyces* species all with dDDH, ANIb, ANIm and TETRA values with their closest phylogenomic neighbour below 70, 95, 95 and 0.999, respectively, following the established practice [48,50]. The novel strains’ phylogenomic relationship is shown in Fig. 2.

**Table 4.** The dDDH, ANIb, ANIm and TETRA values between isolate genomes and the strains of the most closely related *Streptomyces* species

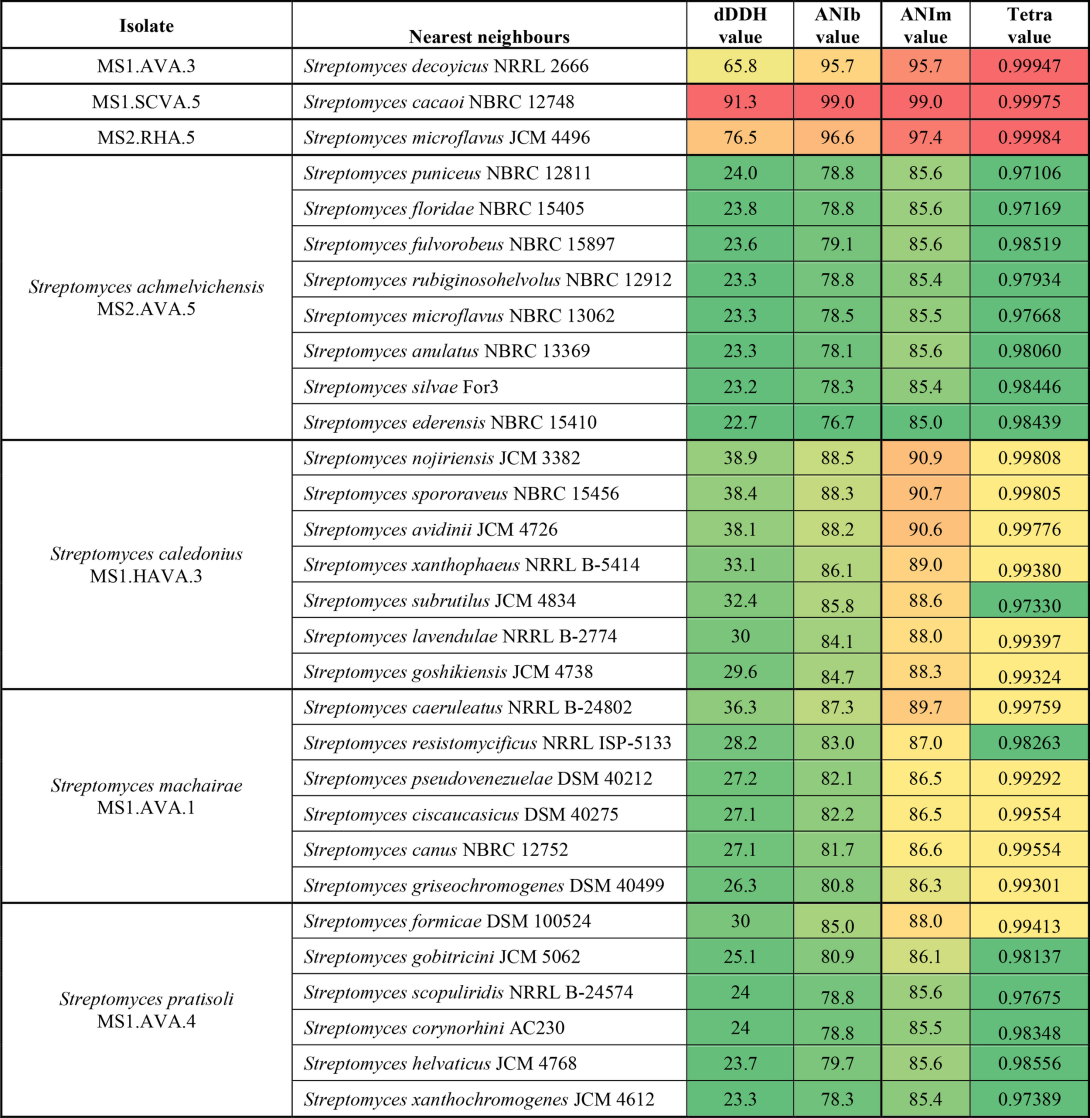 ­

### Phylogeny

**Fig. 3.**
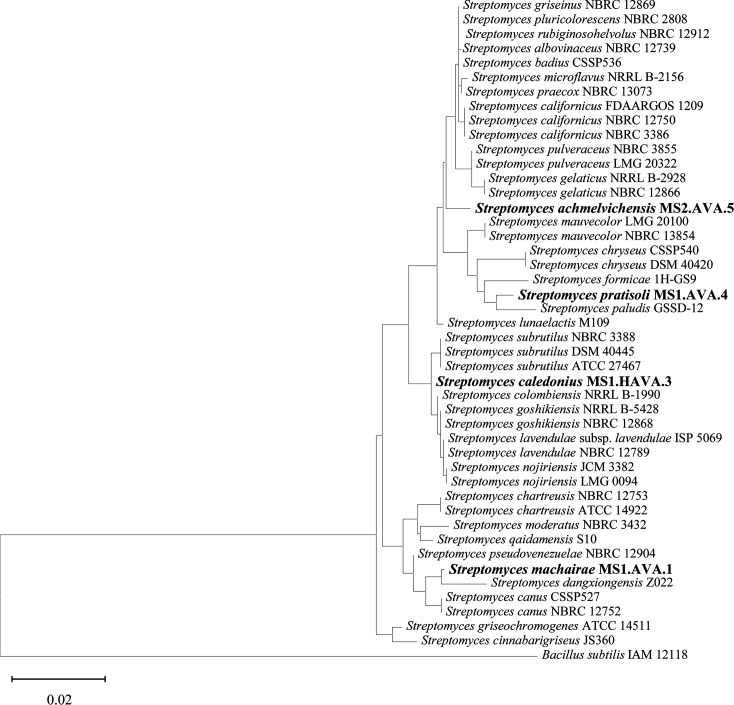
Phylogenetic maximum-likelihood tree based on 16S rRNA gene sequences of *Streptomyces machairae* MS1.AVA.1, *Streptomyces pratisoli* MS1.AVA.4, *Streptomyces caledonius* MS1.HAVA.3 and *Streptomyces achmelvichensis* MS2.AVA.5 (in bold) and representative members of the genus *Streptomyces*. Numbers at branching points represent bootstrap values from 1,000 replications. *B. subtilis* IAM 12118 was used as an outgroup. Bar, 0.02 changes per nt position.

The obtained 16S rRNA gene sequences (ranging from 1,472, MS1.AVA.4, to 1,525 bp, MS2.AVA.5) were compared with entries in public databases using blastn (https://blast.ncbi.nlm.nih.gov/Blast.cgi) with the default settings. A phylogenetic maximum-likelihood tree was constructed based on 16S rRNA gene sequences (Fig. 3).

### Genomic mining: BGCs

**Fig. 4.**
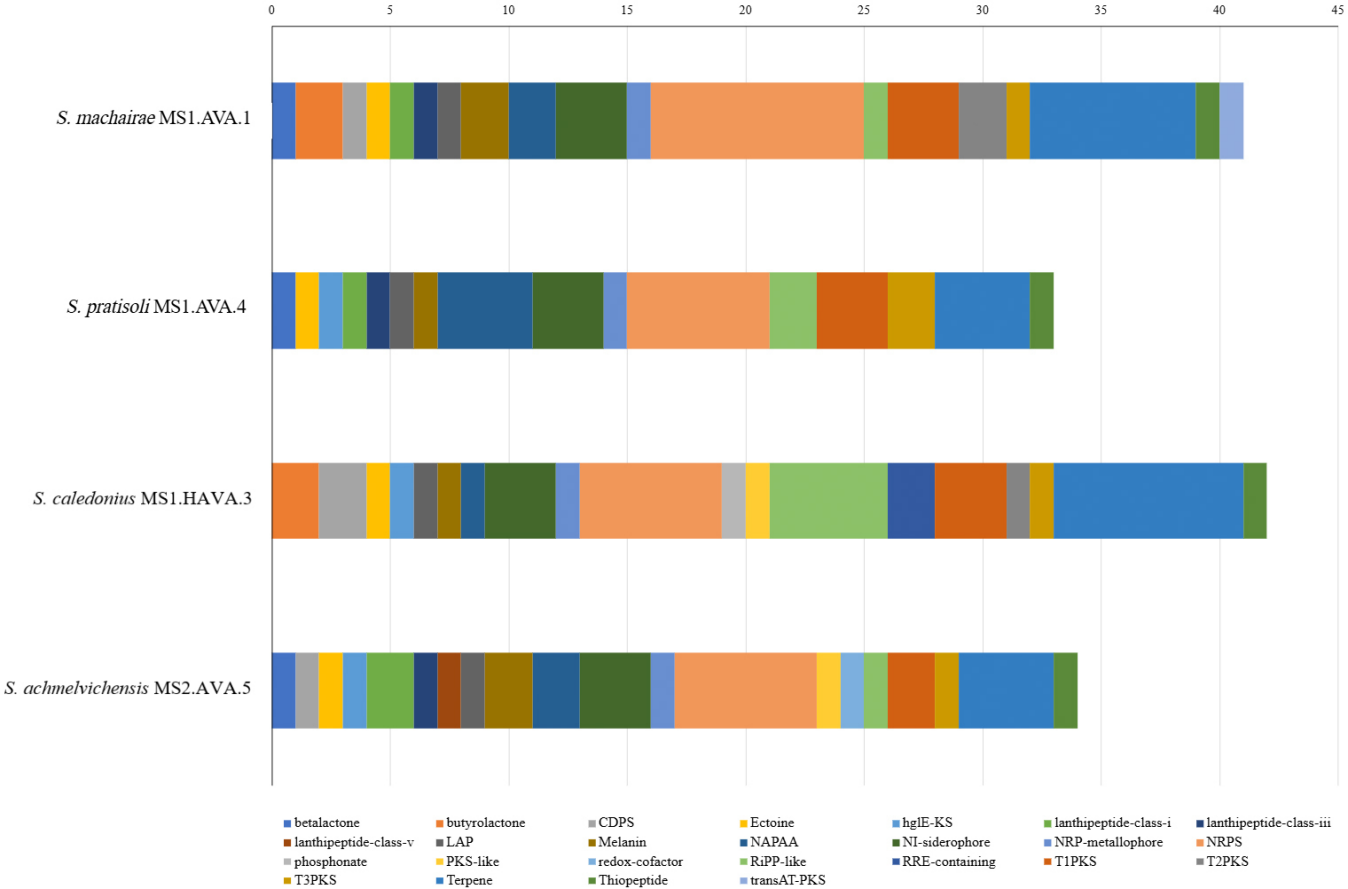
Number and type of BGCs detected in the novel strains.

The number of BGCs found in the genomes of the novel *Streptomyces* strains ranges from 33 in *Streptomyces pratisoli* MS1.AVA.4 to 42 in *Streptomyces caledonius* MS1.HAVA.3 (Fig. 4). Interestingly, the genomes of *Streptomyces achmelvichensis* MS2.AVA.5 and *Streptomyces pratisoli* MS1.AVA.4 contained a similar range of diverse bioclusters. The genomes of all strains were found to have bioclusters predicted to express core specialized metabolites, such as ectoines and melanin pigments, which is in good agreement with previous studies on streptomycetes [70,71]. Again, as in previous studies [35,72], many of the BGCs found in these genomes are predicted to encode for druggable compounds, such as lanthipeptides, non-ribosomal peptide synthases, polyketide synthases, thiopeptides and post-translated modified peptides (RiPP) (Table S6). It is important to focus on BGCs that show low homology (<30%) [73] to their closest neighbour, as this indicates that they are likely to be novel.

The genomes contain BGCs that are predicted to encode for several known compounds, albeit with low levels of similarity (Table S6). The *Streptomyces machairae* MS1.AVA.1 strain, for instance, has the genetic capacity to synthesize antibacterial compounds, which show low homology to belactosin A, an antitumour antibiotic [74]; glycinocin A, a calcium-dependent amphomycin antibiotic effective against *S. aureus* [75,77]; nocardiopsistins, new angucyclines which inhibit MRSA [78]; grincamycin, an isotetracenone antibiotic [79]; and vazabitide A, an amino-group carrier protein used for metabolite synthesis in *Streptomyces* [80]. Additionally, this strain has the capacity to produce an antibiotic with 62% similarity to cahuitamycins A to C, biofilm inhibitor with antibacterial activity that are under investigation as potential drug candidates due to the role of biofilms in antibiotic resistance [81,82].

The strain *Streptomyces pratisoli* MS1.AVA.4 harbours BGCs with low homology (<30%) to known antibacterial compounds, including azicemicin B [83], niphimycins C–E [84] and paromycin [85]. Similarly, the *Streptomyces caledonius* MS1.HAVA.3 strain is predicted to encode for compounds with potential antibacterial activity, such as variochelin A/B [86], auroramycin [87], toxoflavin [88,89], monensin [90] and muramycin C1 [91]. Finally, the *Streptomyces achmelvichensis* MS2.AVA.5 strain produces the antibacterial agents paenibactin [92], himastatin [93], lankacidin C [94] and bombyxamycin A/B [95]. It can be concluded that the novel streptomycetes from the machair grassland soil are a potentially rich source of new specialized compounds.

### Genomic mining: stress-related genes

**Fig. 5.**
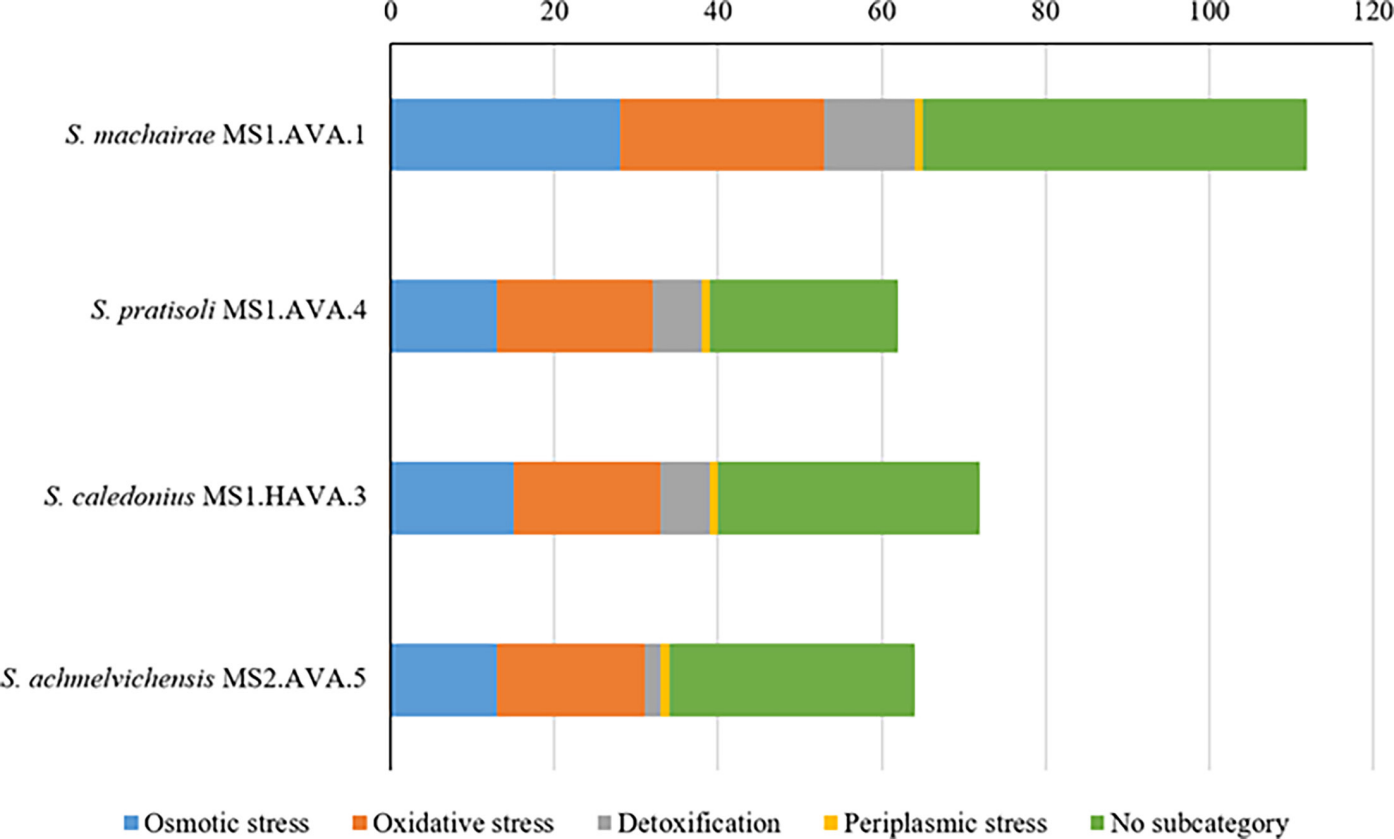
Number and type of stress-related genes detected in the novel strains.

Machair grassland soils are characterized by an alkaline pH, moderately high organic matter content, low phosphate levels, mineral particles composed of calcium carbonate and seasonal variations in rainfall and temperature, all of which influence the composition of actinomycete communities. Consequently, stress-related genes were sought in the genomes to gain insight into how they have adapted to conditions in machair soils. The genomes of all strains were rich in genes associated with detoxification and osmotic and oxidative stress, as shown in Fig. 5. In this context, it is interesting that mineral composition and salinity have been shown to be major environmental factors influencing bacterial communities associated with the soils of coastal sand dune plants [96,97]. Again, the fact that oxidative stress-related genes accounted for nearly 30% of the total number of putative stress-related genes may be a function of the low phosphate levels in machair grassland soils [18]. Genes expressing sigmaB regulation were found to have the highest incidence; these genes are associated with a range of environmental stresses including low and high pH values [98]. The number of stress-related genes ranged from 62 for the novel *Streptomyces pratisoli* MS1.AVA.4 to 112 for the strain of *Streptomyces machairae* MS1.AVA.1. The RAST-SEED web server was also used to generate the subsystem profiles of the isolates, as exemplified in Fig. S6. The latter gave similar profiles, as exemplified by *Streptomyces machairae* MS1.AVA.1, which contains many genes associated with aa metabolism, protein metabolism, carbohydrates, lipids, cofactors, vitamins, prosthetic groups and pigments.

## Concluding statement

It is clear that machair grassland soils are a source of novel streptomycetes, which have the capacity to synthesize a diverse range of specialized metabolites and stress-related compounds. While further research is needed to fully explore the full extent of their biotechnological and ecological value, this highlights the importance of preserving this unique habitat. Currently, the machair grassland at Achmelvich is not designated as a Site of Special Scientific Interest. However, the findings presented here provide justification to reconsider its status, ensuring the conservation of its biodiversity and the protection of actinomycetes with significant biotechnological potential.

## Description of *Streptomyces* sp. nov.

Description of *Streptomyces machairae* sp. nov. *Streptomyces machairae* (ma.chair’ae. N.L. gen. n. *machairae*, of machair, the Gaelic word for grassland soil). Aerobic, Gram-stain-positive actinomycetes that form an extensively branched dark grey substrate mycelium and carries a bluish grey or white aerial spore mass on oatmeal agar. Does not produce melanin pigments on peptone-yeast extract-iron agar. Grows from 4 to 37 °C (optimally at 28 °C), from pH 5.5 to 9.5 and in the presence of up to 90 ppt NaCl (optimally at 10–30 ppt). The genome size of the type strain is 9.4 Mbp and the corresponding G+C content from 70.54%. *Streptomyces machairae* was isolated on 6 June 2023, using arginine-vitamin agar as selective media, from a machair grassland soil at Achmelvich Bay, near Lochinver, Sutherland, Scotland. The type strain is MS1.AVA.1, and the GenBank accession numbers for the 16S rRNA gene and whole-genome sequences are PQ579659 and JBBKAK000000000, respectively. The accession numbers for DSMZ and NCIMB culture collections are DSM118363 and 15553, respectively.

Description of *Streptomyces achmelvichensis* sp. nov. *Streptomyces achmelvichensis* (ach.mel.vich.en’sis. N.L. masc. adj. *achmelvichensis*, of or belonging to Achmelvich, the source of the isolate). Aerobic, Gram-stain-positive actinomycetes that form an extensively branched light brown substrate mycelium, a white aerial spore mass and yellow diffusible pigment on oatmeal agar. Melanin pigments are formed on peptone-yeast extract-iron agar. Grows from 4 to 28 °C, but not at 37 °C, from pH 5.5 to 9.5 and in the presence of up to 70 ppt NaCl. Genome size is 9.6 Mbp and the G+C content 70.04%. *Streptomyces achmelvichensis* was isolated on 6 June 2023, using arginine-vitamin agar as selective media, from a machair grassland soil at Achmelvich Bay, near Lochinver, Sutherland, Scotland. The type strain is MS2.AVA.5, and the GenBank accession numbers for the 16S rRNA gene and whole-genome sequences are PQ579660 and JBBKAJ000000000, respectively. The accession numbers for DSMZ and NCIMB culture collections are DSM118366 and 15556, respectively.

Description of *Streptomyces pratisoli* sp. nov. *Streptomyces pratisoli* (pra.ti.so’li. L. neut. n. *pratum,* a meadow; L. neut. n. *solum,* soil; N.L. gen. n. *pratisoli*, of meadow soil). Aerobic, Gram-stain-positive actinomycetes that form an extensively branched dark brown substrate mycelium, a white aerial spore mass and a dark brown diffusible pigment on oatmeal agar. Melanin pigments are formed on peptone-yeast extract-iron agar. Grows from 15 to 37 °C, from pH 5.5 to 9.5 and in the presence of 10 to 50 ppt NaCl. The genome size of the type strain is 8.6 Mbp and the G+C content 70.79%. *Streptomyces pratisoli* was isolated on 6 June 2023, using arginine-vitamin agar as selective media, from a machair grassland soil at Achmelvich Bay, near Lochinver, Sutherland, Scotland. The type strain is MS1.AVA.4, and the GenBank accession numbers for the 16S rRNA gene and whole-genome sequences are PQ579661 and JBBKAI000000000, respectively. The accession numbers for DSMZ and NCIMB culture collections are DSM118364 and 15555, respectively.

Description of *Streptomyces caledonius* sp. nov. *Streptomyces caledonius* (ca.le.do’ni.us. L. masc. adj. *caledonius*, pertaining to Scotland). Aerobic, Gram-stain-positive actinomycete that forms an extensively branched light brown substrate mycelium and a pinkish white aerial spore mass on oatmeal agar. Does not form melanin pigments on peptone-yeast extract-iron agar. Grows from 15 to 28°C but not at 37 °C, from pH 5.5 to 9.5 and in the presence of up to 90 ppt NaCl (optimally at 10 to 30 ppt). The genome size of the type strain is 8.8 Mbp and the G+C content 71.79%. *Streptomyces caledonius* was isolated on 6 June 2023, using humic acid-vitamin agar as selective media, from a machair grassland soil at Achmelvich Bay, near Lochinver, Sutherland, Scotland. The type strain is MS1.HAVA.3, and the GenBank accession numbers for the 16S rRNA gene and whole-genome sequences are PQ579662 and JBBKAM000000000, respectively. The accession numbers for DSMZ and NCIMB culture collections are DSM118365 and 15554, respectively.

## Supplementary material

10.1099/ijsem.0.006736 Uncited Supplementary Material 1.

## Abbreviations

BGCs: biosynthetic gene clusters
dDDH: dDNA:DNA hybridization
ISP: International *Streptomyces* Project
TG-DSC-QMS: thermogravimetry-differential scanning calorimetry quadrupole with mass spectrometry
TYGS: Type Strain Genome Server
