# Supplementary material for: Identification of four novel Streptomyces isolated from machair grassland soil using a culture-based bioprospecting strategy: Streptomyces caledonius sp. nov., Streptomyces machairae sp. nov., Streptomyces pratisoli sp. nov. and Streptomyces achmelvichensis sp. nov
Source: Int J Syst Evol Microbiol. 2025 Apr 9;75(4):006736. doi: 10.1099/ijsem.0.006736 (PMC12281783; doi:10.1099/ijsem.0.006736)

Supplementary material

Figure S1. Sampled area of Machair grassland at Achmelvich bay, Sutherland, Scotland. Photo by Michael Goodfellow, 25th May 2023.

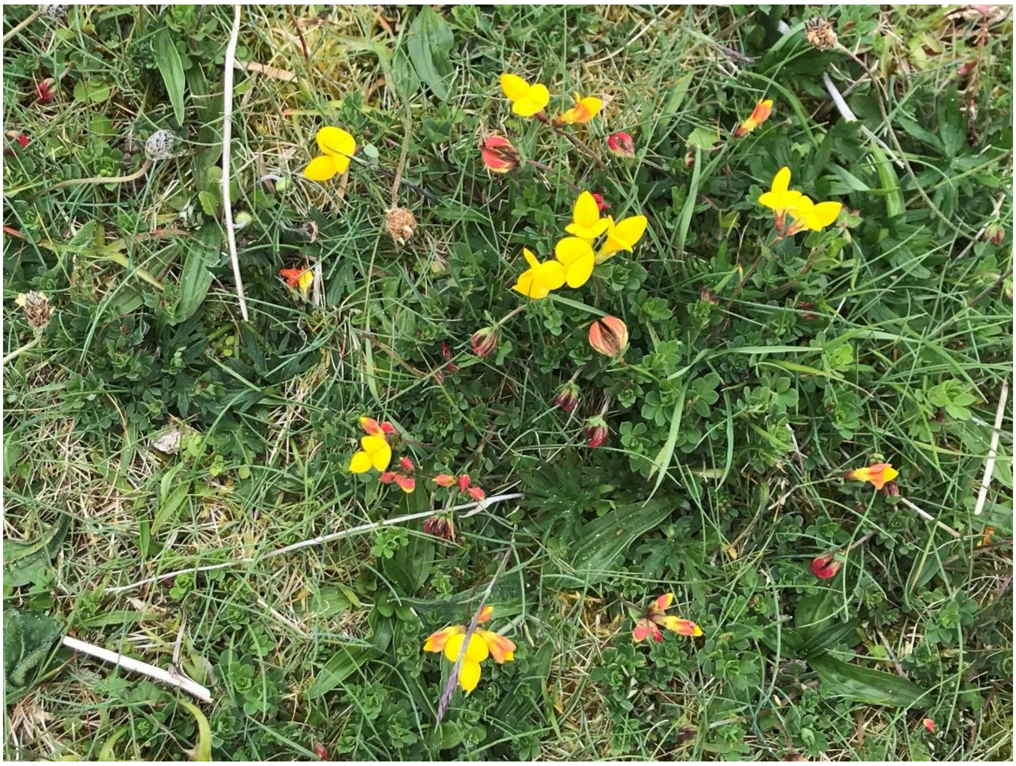

Table S1. *Bacillus subtilis* reporter strains used in plug assays.

Reporter strain	Targets	Positive control
dinB^{CR}	DNA synthesis	Naladixic acid
yjaX^{ER}	Fatty acid synthesis	Triclosan
ypuA^{ER}	Cell envelope synthesis	Cefoxitin
yvgS^{ER}	RNA synthesis	Rifampicin
yvqI^{ER}	Cell wall synthesis	Bacitracin

^{ER}: Erthomycin resistant, ^{CR}: Chloramphenicol resistant.

Table S2. Concentration and purity (A260/A280) of DNA prepared from isolates selected for nanopore sequencing.

Isolate code	Concentration (µg/µl)	A260/A280
MS1.AVA.1	7.70	1.925
MS2.RHA.5	28.50	1.990
MS1.AVA.3	7.85	1.653
MS1.AVA.4	30.50	1.860
MS2.AVA.5	22.10	1.830
MS1.HAVA.3	85.50	1.550
MS1.SCVA.5	9.40	1.664

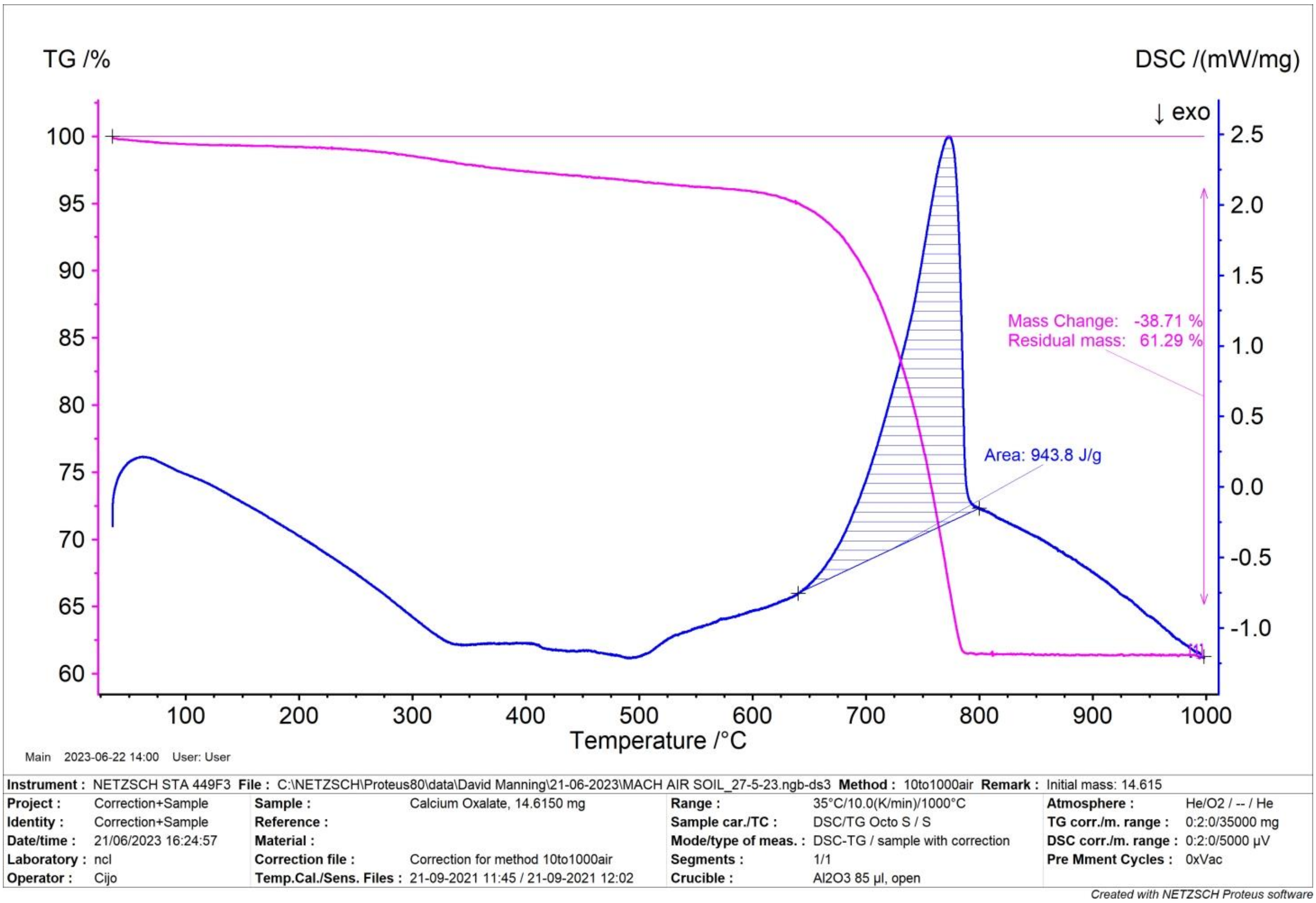

Figure S2. Thermal analysis output, showing weight loss (TG) as a function of temperature of the heating experiment and the DSC (differential scanning calorimetry) signal showing a peak at this temperature range.

Table S3. Assignment of representative isolates to colour-groups. The numbers in parenthesis denotes colour codes taken from the ISCC-NBS charts [30].

Colour groups	Isolate codes**	Number of isolates	Aerial spore mass colour	Substrate mycelium colour	Diffusible pigment
**1**	MS1.AVA.1†; MS1.AVA.2; MS1.HAVA.1; MS1.Gau.2; MS1.HAVA.2; MS1.HAVA.4 MS1.SCVA.2; MS1.SCVA.3;	8	Bluish gray (191)	Black (266)	Black (266)
**2**	MS2.HAVA.2; MS2.AVA.5; MS2.SCVA.2; MS2.RHA.5†; MS2.Gau.4	5	Yellow (87)	White (263)	Dark brown (59)
**3**	MS1.Gau.3; MS1.AVA.3†	2	Light gray (264)	Black (267)	Black (267)
**4**	MS1.AVA.4*†; MS1.AVA.6*	2	White (263)	Dark brown (59)	Dark brown (59)
**5**	MS2.AVA.4*; MS2.AVA.5*†	2	White (263)	Light brown (57)	Yellow (87)
**6**	MS1.HAVA.3†	1	Pinkish white (9)	Light brown (57)	Not detectable
**7**	MS1.SCVA.5†	1	White (263)	Light brown (57)	Not detectable

* Isolates which produced melanin pigments on ISP6 medium after 5 days at 28°C and † those included in the whole-genome sequencing analyses.

** Key to isolate codes: MS1 and MS2, pilot and subsequent isolation from machair soil; AVA, Gau, HAVA, RHA and SCVA isolates from arginine-vitamin, Gause’s, humic acid-vitamin, raffinose-histidine and starch-casein vitamin agars, respectively.

Figure S3. Ability of representative isolates to grow at 4°C, 15°C, 38°C, and 45°C, reading from left hand column to right.

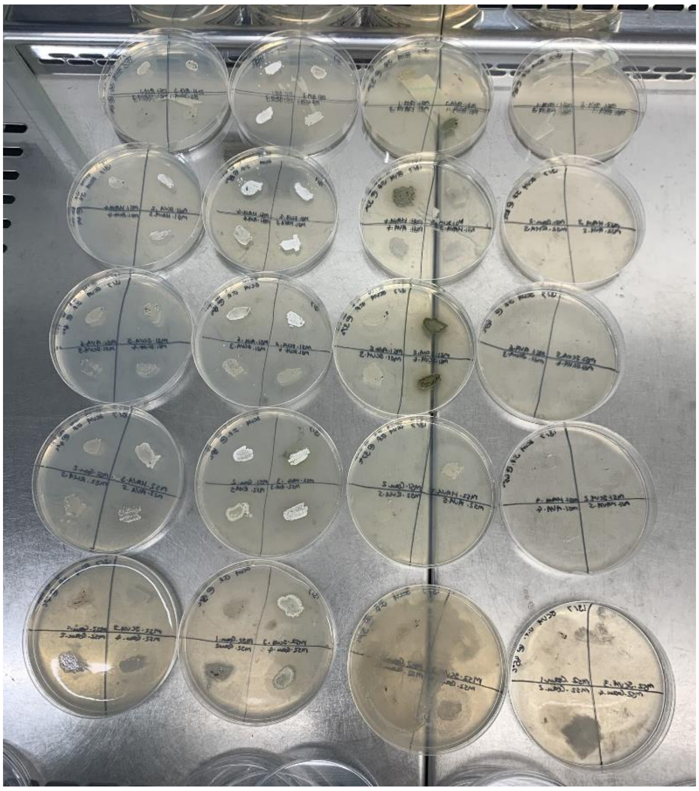

Figure S4. Ability of representative isolates to grow in the presence of 3%, 5%, 7%, and 9% NaCl, reading from left hand column to right.

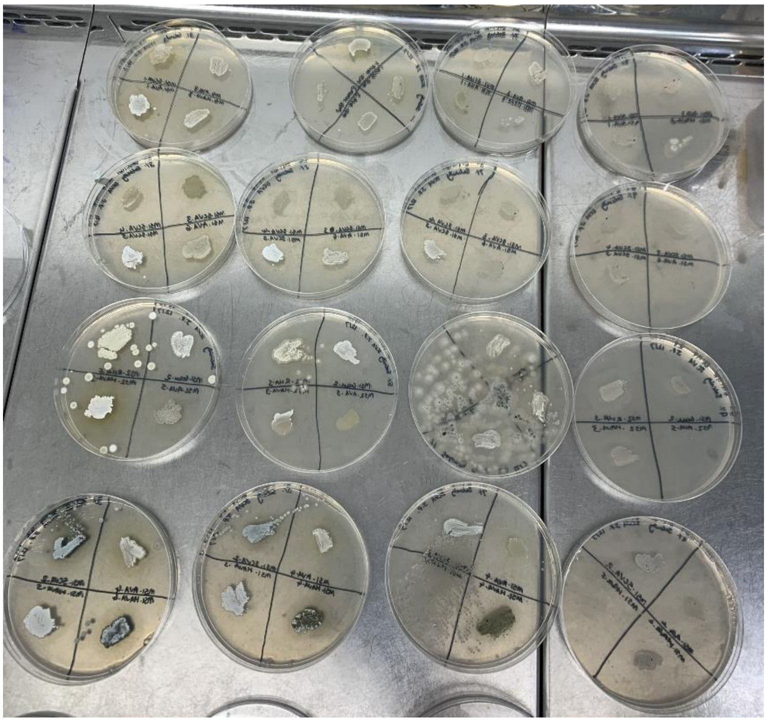

Figure S5. Ability of representative isolates to grow at pH 4.5, 5.5, 6.5, 8.5, and 9.5, reading from left hand column to right.

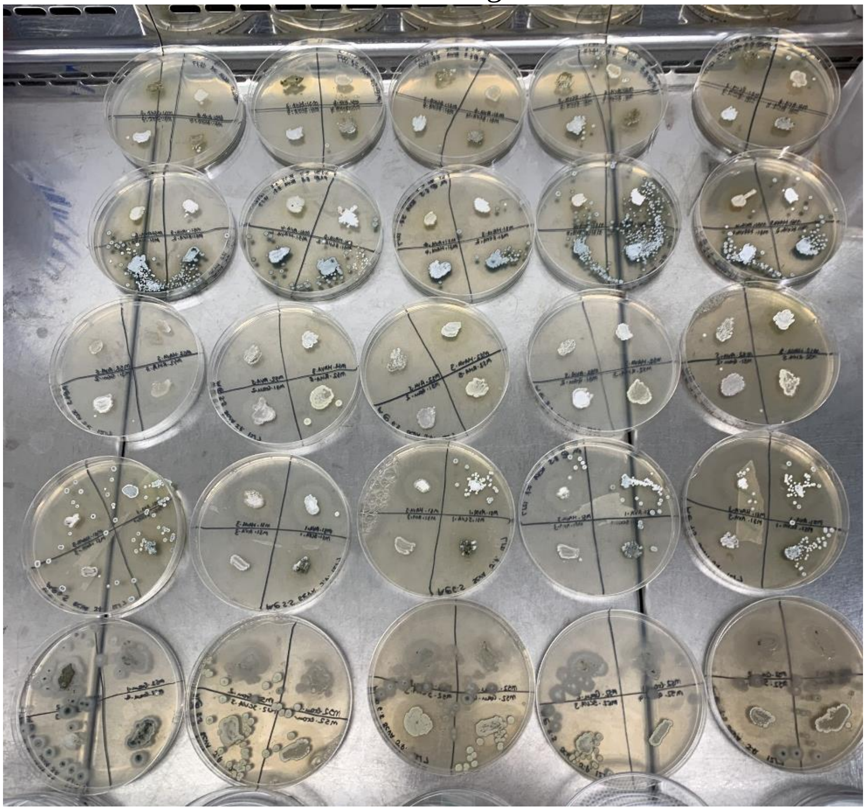

Table S4. Inhibition zones (mm) shown by representative isolates in the plug assays after overnight incubation at 37°C; -, no inhibition.

Isolates	*Escherichia coli*	*Bacillus subtilis*	*Pseudomonas fluorescens*	*Micrococcus luteus*	*Saccharomyces cerevisiae*
MS1.AVA.1	-	-	-	24	14
MS2.RHA.5	-	12	-	14	-
MS1.AVA.3	-	-	-	18	20
MS1.AVA.4	-	-	-	-	24
MS2.AVA.5	-	8	-	-	18
MS1.HAVA.3	-	-	-	20	14
MS1.SCVA.5	-	8	22	-	-
Percentage of isolates giving positive hits (%)	0.0	42.9	14.3	57.1	71.4

Table S5. Isolates showing activity against the *B. subtilis* reporters irrespective of whether blue halos were produced. Numbers indicate zones of inhibition (mm); -, no inhibition; [blue shading], formation of blue halos.

Colour groups	Isolate	*Bacillus subtilis* mutants				
		yvqI	*yvgS*	*ypuA*	*yjaX*	*dinB*
1	MS1.AVA.1	7	7	-	-	7
2	MS2.RHA.5	7	-	7	7	7
3	MS1.AVA.3	7	9	7	7	7
4	MS1.AVA.4	-	-	-	-	7
5	MS2.AVA.5	-	-	7	7	-
6	MS1.HAVA.3	9	11	11	13	11
7	MS1.SCVA.5	-	-	-	-	-
Percentage of isolates producing blue halos (%)		42.9	28.6	14.3	42.9	42.9

Table S6. The type and closest known compounds of biosynthetic gene clusters from novel strains, and their percentage similarity to closest known compounds.

Isolate	Type	Closest known compound	Similarity
***Streptomyces machairae* MS1.AVA.1**	NRPS,T1PKS	prunipeptin	100%
	T1PKS	4-hexadecanoyl-3-hydroxy-2-(hydroxymethyl)-2H-furan-5-one	90%
	T2PKS,butyrolactone	maduralactomycin A/maduralactomycin B/actinospirol A/actinospirol B	72%
	NRP-metallophore,NRPS	cahuitamycin A/cahuitamycin B/cahuitamycin C	62%
	NAPAA	belactosin A/belactosin C	29%
	NRPS	glycinocin A	9%
	CDPS	nocardiopsistin A/nocardiopsistin B/nocardiopsistin C	9%
	NI-siderophore	grincamycin	8%
	betalactone	vazabitide A	6%
	terpene	cyphomycin	2%
***Streptomyces pratisoli* MS1.AVA.4**	arylpolyene,T1PKS,NRPS-like,RiPP-like	o-dialkylbenzene 1/o-dialkylbenzene 2	22%
	T1PKS,NRPS	azicemicin B	11%
	thiopeptide,LAP	niphimycins C-E	6%
	NRPS-like	paromomycin	5%
	NAPAA	paromomycin	5%
***Streptomyces caledonius* MS1.HAVA.3**	T2PKS,RiPP-like	lomaiviticin A/lomaiviticin C/lomaiviticin D/lomaiviticin E	55%
	phosphonate	fosfazinomycin A/fosfazinomycin B	47%
	T1PKS,PKS-like,amglyccycl,butyrolactone	sceliphrolactam	44%
	RRE-containing	fosfazinomycin A/fosfazinomycin B	36%
	NRPS-like,NRPS,T1PKS	variochelin A/variochelin B	30%
	T1PKS	auroramycin	22%
	NRPS	omnipeptin	20%
	NI-siderophore	K-252a	14%
	terpene	toxoflavin/fervenulin	7%
	terpene	monensin	5%
	NRPS,NRPS-like	muraymycin C1	5%
	CDPS	deoxyhangtaimycin	2%
***Streptomyces achmelvichensis* MS2.AVA.5**	NRP-metallophore,NRPS	scabichelin	100%
	NRPS	paenibactin	66%
	hglE-KS,T1PKS,PKS-like	nataxazole	62%
	other,NRPS,betalactone	himastatin	36%
	NAPAA	colibrimycin	19%
	CDPS	BD-12	17%
	redox-cofactor	lankacidin C	13%
	LAP,thiopeptide	bombyxamycin A/bombyxamycin B	11%

Figure S6. An example of the subsystem profiles generated by the RAST-SEED webserver (https://rast.nmpdr.org//).

S. machairae
MS1.AVA.1

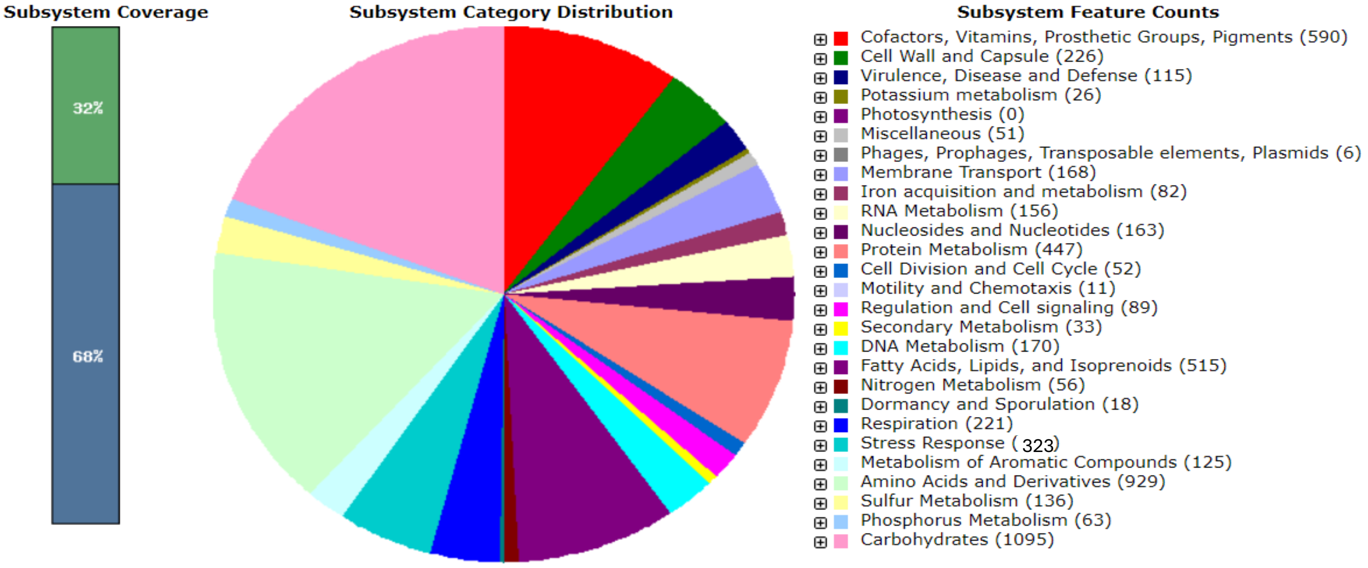

Supplement: Uncited Supplementary Material 1. [file ijsem-75-06736-s001.pdf]
